# The Complex Role of MAPK/ERK Signaling Pathway in Different Types of Thrombocytopenia

**DOI:** 10.3390/cimb47110960

**Published:** 2025-11-19

**Authors:** Peipei Xue, Maoshan Chen

**Affiliations:** 1 Laboratory of Radiation Biology, Department of Blood Transfusion, Laboratory Medicine Center, The Second Affiliated Hospital, Army Medical University, Chongqing 400037, China; xuepeipei@alumni.hust.edu.cn; 2 Laboratory of Precision Medicine, Department of Blood Transfusion, Laboratory Medicine Center, The Second Affiliated Hospital, Army Medical University, Chongqing 400037, China; 3 Hematopoietic Acute Radiation Syndrome Medical and Pharmaceutical Basic Research Innovation Center, Ministry of Education of the People’s Republic of China, Department of Blood Transfusion, The Second Affiliated Hospital, Army Medical University, Chongqing 400037, China

**Keywords:** thrombocytopenia, ERK/MAPK signaling pathway, inhibitors

## Abstract

Thrombocytopenia is a common hematological disorder caused by a variety of factors. It often complicates diseases and treatment options for this condition are quite limited. There are no approved drugs for certain types of thrombocytopenia. Therefore, understanding the mechanisms of thrombocytopenia is crucial for drug selection and development. The ERK/MAPK signaling pathway plays a significant yet complex role in the formation of megakaryocyte–platelet differentiation and reports on its function are inconsistent. It may be activated, inhibited, or unaffected in different types of thrombocytopenia models. Several drugs targeting the ERK/MAPK signaling pathway have been used for thrombocytopenia in clinical settings, and some small molecule inhibitors have also shown potential therapeutic efficacy for thrombocytopenia through this pathway. In this review, we will summarize both historical and new evidence regarding the roles of the ERK/MAPK signaling pathway in various types of thrombocytopenia and discuss current therapies and future treatment strategies.

## 1. Introduction

Platelets are the smallest blood cells, typically ~2 μm in diameter and enucleated in the body and are produced by megakaryocytes (MKs) in the bone marrow [1,2]. They have been reported to play important roles in innate immune response, hemostasis, angiogenesis, thrombosis, and infection [1,2]. Thrombocytopenia is a disease with a low platelet count below the lower limit (e.g., 150 × 10^9^/L for adults) [3]. It has been a clinical challenge for a long time and can lead to infection, abnormal bleeding, and even death [3]. There are some major types of thrombocytopenia including immune thrombocytopenia (ITP), hereditary thrombocytopenia, tumor and tumor therapy-induced thrombocytopenia, thrombosis-induced thrombocytopenia, and inflammation and infectious-induced thrombocytopenia. Unfortunately, current treatment options for this disease are limited [1].

In clinics, nonspecific drugs such as hormones, cytokines, immunosuppressive agents, and platelet transfusion are normally used. Although platelet transfusion is an effective treatment for severe thrombocytopenia, there is a risk of causing hemolytic reactions when ABO-mismatched, transfusion-related acute lung injury occurs due to a large amount of plasma from a single donor, and infectious diseases can be transmitted due to fewer donor exposures [4,5]. For chronic thrombocytopenia patients who do not respond to steroids, thrombopoietin receptor (TPOR) agonists are the primary second-line option [6]. However, TPOR agonists are not suitable for patients who have a complete loss *c-MPL* (C-mannosylation of thrombopoietin receptor) or refractory to these agonists [7,8,9]. These agonists may increase the risk of acute myelogenous leukemia, venous and arterial thrombosis, and liver toxicity. The situation of rapid treatment of radiotherapy or chemotherapy-induced thrombocytopenia is even worse and currently there are no approved drugs specific for these patients [10]. Therefore, understanding the mechanisms of thrombocytopenia is important for drug selection and the development of thrombocytopenia treatment.

The MAPK/ERK (mitogen-activated protein kinase/extracellular signal-regulated kinase) signaling pathway is crucial in hematological research [1,2]. However, it exhibits contrasting characteristics during megakaryocyte–platelet formation: on one hand, it is activated by the TPO signaling pathway which leads to positively driven megakaryocyte maturation and platelet production [11]; on the other hand, it acts as a key effector in platelet activation processes, leading to activated platelets being cleared by the immune system [12]. Therefore, systematically elucidating the role of the ERK/MAPK pathway in different states of platelets has a significant theoretical value for understanding the multifaceted pathogenesis of thrombocytopenia. Furthermore, it may provide a crucial scientific rationale for developing novel therapeutic strategies targeting this pathway. This review will focus on this core objective, aiming to summarize evidence of its pluripotent in thrombocytopenia causes and analyze its pathological contributions and therapeutic potential across various forms of thrombocytopenia.

## 2. The Role of MAPK/ERK Signaling Pathway in the Formation of MK-Platelet Differentiation

The MAPK signaling is an evolutionarily conserved signal transduction pathway (e.g., transiting extracellular signals into the cytoplasm) and is involved in the cellular growth, differentiation, and survival of proliferative cells. There are 14 MAPKs in the human genome participating in multiple phosphorylation cascades. They belong to three major functional families: ERKs, JNKs, and p38 [13]. Among them, ERK1/2 has been well studied in the hematological system. It has been shown that ERK1/2 plays a pivotal role in platelet disorders, including thrombocytopenia [14]. The controversy of the functions of the ERK/MAPK signaling pathway in MK-platelet differentiation was mainly about the small molecule compounds of ERK (Table 1).

### 2.1. Evidence Supporting a Positive Role of ERK/MAPK in Megakaryopoiesis

In 1997, Racke and colleagues first reported that sustained activation of ERK is required for MK differentiation [15]. In the same year, Melemed confirmed that an activated MAPK pathway was sufficient and necessary to induce differentiation of the MK lineage [16]. ERK signaling can be activated by thrombin and phorbol ester (PMA) through the activation of the protein kinase C in a concentration-dependent manner and the translocation of ERK1 and ERK2 can be promoted from cytosol to cytoskeleton [17]. Using K562 cell models, Levay and colleagues found that PMA increased the level of phosphorylating ERK (p-ERK) and led to the favor of both differentiation and polyploidization of MKs [18]. In addition, the activation level of ERK1 and ERK2 was reported to be a possible key factor for cell fate determination toward erythroid and megakaryocytic lineages of a human leukemia cell line (UT-7/GM) [19]. The requirement of ERK activity may vary during different aspects of MK differentiation and the protein tyrosine phosphatase HepTP has been reported to regulate the nuclear translocation of ERK2 and modulate its expression in the differentiation of K562 cells [20].

Succinate, also known as succinic acid in its protonated form, was found to activate the MAPK/ERK pathway and enhance the platelet level in a chemotherapy-induced myelosuppression mouse model [21]. Both MEK (e.g., U0126 and PD184161) and ERK (e.g., PD98059) inhibitors can reduce the megakaryocytes’ ploidy and platelet formation and alter the expression of differentiation markers like CD10, CD44, CD41, and CD61 [22,23,24]. Other platelet therapy-related metabolites and traditional Chinese medicine monomers also indicated that high-dose R-lipoacid administration reduced rat platelet counts and the phosphorylation level of ERK significantly [25]. The human milk oligosaccharide (HMO) 3′-sialyl lactose can also induce megakaryocyte differentiation through the activation of ERK [26]. Di Buduo et al. revealed that eltrombopag promoted human cell’s megakaryopoiesis dependent on the activation of the AKT/ERK pathway [27].

### 2.2. Studies Reporting Neutral or Inhibitory Roles

However, there are some different voices. Mazharian et al. found that the activation of ERK-1/-2 signaling molecules induces the proliferation of immature megakaryocytes, but not the production of platelets [28]. Shelly et al. showed that PMA-induced multinucleated cells of K562 cells were independent of the MAPK pathway and internalization of PMA transferrin receptor was not regulated by the MAPK pathway [29]. Further, Kalina et al. found that MAPK was highly expressed in patients with refractory anemia, hyperblastic transformation, and acute myeloid leukemia, but its phosphorylation level was not elevated or even undetectable [30]. Ito et al. showed that the ERK/MAPK signaling pathway was independent of MK proliferation and differentiation [31]. Bluteau et al. suggested that sustained Ankrd26 expression caused sustained ERK activation leading to impaired proplatelet formation, reduced with polyploidy [32]. STI571 induced megakaryocyte (CD41a and CD42) markers of K562 cells and induced dephosphorylation of extracellular signal-regulated kinase (ERK) in its cells [33].

### 2.3. Possible Mechanistic Explanations for These Differences

The reason why the MAPK/ERK signaling pathway showed different roles in the formation of MK-platelet differentiation is due to varying levels of ERK activation. Research indicated that transient or acute MAPK activation, which occurs in the cell cytoplasm (minutes to hours) is typical of cell proliferation, whereas sustained MAPK activation into the nucleus (lasting many hours or several days) is characteristic of cell-cycle arrest and differentiation [34].

**Table 1:** The role of the ERK/MAPK signaling pathway in megakaryocyte-platelet differentiation.

Signal Pathway	Cell Function	Correlation	References
ERK sustained activation	Megakaryocyte differentiation	+	[15]
MAPK pathway activation	Megakaryocyte differentiation	+	[16]
p-ERK increasing	Differentiation and ploidization of K562 and HEL cells	+	[18,20]
ERK activation	Cells differentiate to become erythroid or megakaryocytic	+	[19]
MAPK/ERK pathway activation	Mouse platelet levels	+	[21]
MEK and ERK activation	Megakaryocytes ploidy and platelets formation	+	[22,23,24,25]
ERK activation	Megakaryocyte differentiation	+	[26]
ERK activation	Human cells megakaryopoiesis	+	[27]
ERK 1/2 activation	Immature megakaryocytes proliferation	−	[28]
MAPK pathway was not regulated	Megakaryocyte differentiation of K562	−	[29]
Phosphorylation of MAPK pathway was not elevated	Myelodys-plastic syndromes	−	[30]
ERK inhibited	MK polyploidy formation enhanced	−	[35]
ERK sustained activation	MK polyploidy and proplatelet formation decreasing	−	[32]
ERK dephosphorylation	K562 megakaryocyte markers increasing	−	[33]

+: positive correlation, −: negative correlation.

## 3. Aberrant MAPK/ERK Signaling Pathway and Thrombocytopenia

### 3.1. Immune Thrombocytopenia

ITP is an autoimmune hemorrhagic disease in which hyperactivation of T cells is critical in its pathogenesis. Using the ITP rat model, miR-557 was identified as a specific miRNA associated with both ITP and TPO treatment [36]; further, the authors found that the miR-557 inhibitor improved ITP by regulating apoptosis-related genes (Bcl2, Casp3, and Bax) and activating the Akt/ERK pathway.

The involvement of MAPK/ERK in ITP is mainly reported by treatment and drug-related studies. For example, eltrombopag, a nonpeptide thrombopoietin receptor agonist used for ITP, induced immature MK proliferation rather than platelet production through the unbalanced activation of AKT and ERK1/2 signaling molecules [27]. Through the activation of the MAPK/ERK and PI3K/Akt signaling pathway, Sijunzi Granules (SGs) were proved to increase the levels of 5-Hydroxytryptamine and its receptors to promote the formation and release of platelets and reduce the incidence and severity of intestinal bleeding in zebrafish [37].

Toll-like receptor 3 (TLR3) agonists can also stimulate MK activation through increasing the protein expression levels of NF-κB, PI3K/Akt, ERK1/2, and p38 pathways; however, it can also result in reduced platelet production in vitro [38]. Additionally, protein kinase C (PKC) has been recognized as an important regulator of megakaryopoiesis; however, the PKCε null mice had higher rebounds thrombocytosis and recovered faster than their littermate *wt* control mice, probably due to the enhanced signal pathway of p-Akt and p-ERK1/2 [39]. Furthermore, Atorvastatin (AT) is an immunomodulatory medication used to lower cholesterol and triglyceride levels to help prevent heart disease, a molecular mechanism is not even fully understood. Xu et al. found that AT can significantly inhibit cell proliferation, lead to cell-cycle arrest, induce cell apoptosis, and inhibit the activation of CD4^+^ T cells [40]. Furthermore, phosphorylation expression of mammalian target of rapamycin (mTOR), protein kinase B (AKT), extracellular signal-regulated kinase (ERK), and activation of rat sarcoma virus (RAS) were significantly reduced after AT treatment in vitro [40]. Similarly, the pathophysiology mechanism of thienopyridine platelet antagonist ticlopidine in the development of thrombotic thrombocytopenic purpura (TTP) is also unclear [41]. The plasma of ticlopidine-linked TTP patients induced hepatic microvascular endothelial cell (MVEC) apoptosis through the prolonged induction of ERK1/2 and p38 in TTP-susceptible MVECs and inhibitors of ERK-1/2 and p38 phosphorylation can abolish this phenomenon [41].

The reason why the ERK/MAPK signaling pathway shows different role in ITP models is because ITP primarily affects immune cells, and platelets themselves can bound with these cells, modulating their phenotype and function. ERK/MAPK signaling may not only regulate megakaryocyte differentiation and platelet production but could also influence platelet–immune cell interactions. Additionally, platelets in ITP patients are often heterogeneous, which may affect how these cells activate ERK/MAPK pathways in immune cells [42]. For example, platelets have been reported to interact with B cells, promoting their differentiation into plasma cells and increasing antibody production [43]. It is plausible that ERK/MAPK signaling could contribute to this platelet-B cell interaction, given its established role in regulating cellular proliferation, differentiation, and activation.

### 3.2. Inherited Thrombocytopenia

Inherited thrombocytopenia is a heterogeneous disorder with germline mutations that influences megakaryopoiesis and platelet biogenesis [44].

Src-related thrombocytopenia (SRC-RT) was reported to be associated with the p.E527K heterozygous germline mutation which leads to the gain of a function variant of Src and inherited platelet disorder [45]. In a family with this germline mutation, four out of seven carriers showed recurrent infections and immune defects, and they not only have problems in platelet production but also have increased platelet consumption and immune dysregulation [45]. Thrombocytopenia 2 (THC2) is another inherited platelet disorder that affects the number of platelets in the blood. Reported THC2 cases are caused by the changes in the ANKRD26 gene [46]. The monoallelic mono nucleotide c.3G>A and c.105C>G were found in the 5′ end of the ANKRD26 gene and could result in the synthesis of N-terminally truncated ANKRD26 isoforms that are stable and functional in cells and significantly activate MAPK/ERK signaling.

### 3.3. Tumor and Tumor Therapy-Induced Thrombocytopenia

#### 3.3.1. Hematologic Malignancies Induced Thrombocytopenia

Hematopoiesis and lineage orientation are regulated by several conserved cellular signaling pathways, including MAPKs [47], and thrombocytopenia is one of the most common symptoms in patients with cancers (see Table 2) [48] like leukemia. Large granular lymphocyte (LGL) leukemia is a rare cytotoxic lymphoproliferative chronic lymphocytic disease and the clonal proliferation of LGL can be stimulated by several molecular pathways including RAF1-MEK1-ERK, JAK-STAT3, PI3K/AKT, and NF-KB [49]. In chronic myeloid leukemia (CML), the cancer’s development and progression and erythropoiesis can be regulated by CT10 regulation of kinase-like (CRKL) (not CRKII) and miR-429 via the activation of the Raf/MEK/ERK pathway [50].

The inhibition of MyoD Family A (I-MFA), a tumor-suppressor gene and transcriptional repressor, was found to regulate megakaryocyte lineage commitment and terminal differentiation [51]. In I-MFA^−/−^ mice, the megakaryocyte/erythroid progenitor differentiation was reduced and the mice showed severe thrombocytopenia when the p-JNK and p-ERK signaling pathways were enhanced [51]. In juvenile myelomonocytic leukemia (JMML), a malignant myeloproliferative neoplasia manifests as splenomegaly with consequential thrombocytopenia; mutations in the PTPN11 (SHP2) gene leads to the hyperactivation of ERK and AKT signaling pathways [52,53]. Using GOF Shp2-bearing cells, bruton tyrosine kinase (BTK) was hyperphosphorylated and promoted PI3K to cooperate with its catalytic subunit of p110δ through a B cell adaptor; dual inhibition of BTK and p110δ can reduce the activation of ERK and AKT [52]. In a mouse model of human JMML, unique anemia and thrombocytopenia were reversed when both BTK and p110δ were inhibited, rather than monocytosis and splenomegaly [52]. The JMML model mouse (Flt3Cre + KrasG12D mice) animals expressing Kras (G12D) in multipotent progenitors and utero are born at normal Mendelian ratios, develop thrombocytopenia, hepatosplenomegaly, and anemia, and die of neonatal myeloid disease [54]. These mice have hematopoietic stem and progenitor cell populations in the BM and spleen, which are hypersensitive to granulocyte-macrophage colony-stimulating factors (GM-CSFs) due to hyperactive RAS/ERK signaling [54].

#### 3.3.2. Solid Tumors Therapy-Induced Thrombocytopenia

Thrombocytopenia is also one of the most common complications during solid tumors treatment (see Table 2). Cancer patients often develop symptoms of thrombocytopenia during chemoradiotherapy. The MAPK/ERK signaling pathway is expressed differently in cancer patients with concomitant thrombocytopenia. The effects of interleukin-2 (IL-2) and recombinant human thrombopoietin (rhTPO) on a basic carboplatin (GC) plus gemcitabine (GEM) treatment regimen in a murine lung carcinoma model was investigated with 50 nude mice with subcutaneous tumors derived from human lung cancer cells with intraperitoneal injection [55]. The results indicated that the supplementation of IL-2 and rhTPO to a GC regime significantly reduced the tumor sizes and increased the platelet count via decreasing the expression levels of p-AMPK, p-AKT, p-ERK, p-PI3K, and RAS1. Ricin induced the activation of ERK, JNK, and p38 MAPK signaling in RAW 264.7 macrophages in vitro as well as in mice kidney cells in vivo, and presented the features of hemolytic uremic syndromes including acute renal failure, thrombocytopenia, and hemolytic anemia [56]. A phase I clinical trial examined the efficacy, safety, and molecular effects of the combination therapy of tesirofenib and sorafenib in patients with advanced melanoma and identified that this combination therapy resulted in dose-limiting toxicities included thrombocytopenia while the p-ERK was not inhibited [57]. In addition, a phase II study of Navitoclax (ABT-263) pretreated for recurrent epithelial ovarian cancers showed that thrombocytopenia was the major side effect of ABT-263 in recurrent epithelial ovarian cancer, but the poor monotherapy activity of ABT-263 was not related to the expression of p-ERK [58]. Furthermore, chemotherapy with cisplatin (cis-diamide dichloroplatin II, one of the most widely used anti-tumor drugs) is usually accompanied by adverse side effects such as thrombocytopenia [59]. Although cisplatin does not induce platelet activation, it significantly impairs platelet function and blocks the ERK pathway.

### 3.4. Thrombosis-Induced Thrombocytopenia

Antiphospholipid syndrome (APS) is a multisystemic autoimmune disorder that can occur in isolation or as part of an underlying systemic autoimmune disorder and is characterized by thrombotic events [60,61]. A considerable number of patients with IgA anti-B2GPI (~0.5% of the SLE patients) showed non-classical features like thrombocytopenia and livedo reticularis [60]. Further, Naranjo and colleagues found that APS patients, including SLE patients with thrombotic events those positive for B2-CIC, which are circulating immune complexes (CIC) formed by beta-2-glycoprotein-I (B2GP1) and anti-B2GP1 antibodies, had lower platelet count and complement levels than those who were negative, suggesting a greater degree of platelet activation [61].

In the view of thrombocytopenia as a feature of APS, which activates antiphospholipid antibodies (aPL) may play a pathogenic role in thrombosis by binding to platelets which may further cause platelet activation and aggregation [62]. In 2004 Vega-Ostertag and colleagues proved that aPL induces the production of thromboxane B_2_ in the presence of subactivating doses of thrombin, mainly through the activation of p38 MAPK and subsequent phosphorylation of cPLA_2_ [62]. This process seems to not involve the ERK-1/2 pathway, at least in the early stages of aPL-mediated platelet activation.

### 3.5. Inflammation and Infectious-Induced Thrombocytopenia

The platelet number can be affected by multiple factors such as aseptic inflammation and infection, and different inflammatory states can cause either thrombocytosis or thrombocytopenia [63]. Tumor necrosis factor α (TNFα) is an important inflammatory cytokine, which can significantly impact MKs via its TNFα receptor 1 and regulate the MAPK-ERK1/2 signaling pathway [63]. An in vivo study involving Balb/c mice demonstrated that TNFα influenced short-term platelet recovery in a dose-dependent manner following bone marrow transplantation [63]. Another important anti-inflammatory cytokine, IL-37, was reported to play a functional role in the regulation of the monocytes/macrophages of ITP patients via decreasing the phosphorylation of the AKT, MAPK, and NF-κB signaling pathways [64]. In addition, IL-37 restored the balance between activation and inhibition of FcγRs and reduced monocyte/macrophage platelet phagocytosis mediated by the antibody.

To our surprise, viral particles were seen inside the platelets and platelet activation markers were detected in patients with viremia [65]. To understand the interactions between these virion and platelets, HIV-1 pseudovirions were injected into Arf6 (ADP-ribosylation factor 6)-/- mice which led to virion uptake, platelet activation, platelet–leukocyte aggregate formation, and mild thrombocytopenia through IKK and AKT activation [65]. Another example is the bovine viral diarrhea virus (BVDV). Acute infection by BVDV led to immune dysfunction and further caused lymphocyte apoptosis and peripheral blood lymphopenia. The authors established a BALB/c mouse model of acute BVDV infection with cytopathic (CP) BVDV (strain NADL) and non-cytopathic (NCP) BVDV (strain NY-1). They observed that both CP and NCP BVDV-infected mice developed leukocytopenia, lymphocytopenia, and thrombocytopenia at day 7 post-infection. They reported that the PD-1 blockade inhibited peripheral blood lymphocyte (PBL) apoptosis and viral replication and increased p-ERK expression in BVDV-infected mice [66]. This research shows that virus infections induce thrombocytopenia through inhibiting the ERK/MAPK signaling pathway.

## 4. Drug Basis on ERK/MAPK Signaling Pathway for the Treatment of Thrombocytopenia Disease

As is shown above, the ERK/MAPK signaling pathway plays an important role in thrombocytopenia although reports on its function are inconsistent. In clinics, several drugs used for thrombocytopenia were found to work via the ERK/MAPK signaling pathway, and some small molecule inhibitors also showed potential therapeutic ability for thrombocytopenia via the ERK/MAPK signaling pathway (Table 3).

### 4.1. Approved Therapies

There are five drugs that have been launched to treat thrombocytopenia in clinics. Shengxuexiaoban capsules, a traditional Chinese medicine classical prescription with the effects of clear heat and detoxifying the blood, cooling the blood, and stopping bleeding, can be used to treat primary ITP through the PI3K-AKT and MAPK-ERK1/2 signaling pathway [67]. Ticlopidine is a thienopyridine platelet antagonist which is associated with thrombotic thrombocytopenic purpura (TTP). Ticlopidine and ticlopidine-TTP patients decreased the transcription level of the ECM component, thrombospondin-1, in microvascular endothelial cells and prolonged the expression of ERK1/2 and p38 in TTP susceptible MVECs [41]. The overexpression of ERK1/2 and p38 induces apoptosis of MVECs, but not of vessel endothelial cells. Therefore, ERK1/2 and p38 may help to elucidate the mechanisms of ticlopidine. Eltrombopag is a nonpeptide thrombopoietin receptor agonist which is used to treat thrombocytopenia caused by various aetiologies such as hepatitis C virus-related thrombocytopenia, immune thrombocytopenia, and aplastic anemia [27]. The AKT and ERK1/2 signaling pathways were crucial for eltrombopag to regulate physiologic thrombopoiesis [27]. As a monoclonal antibody, eculizumab could promptly recover the number of platelets of a thrombocytopenia patient by controlling the complement system’s hyperactivation; however, kidney function did not recover, probably due to the short treatment duration [68]. Hetrombopag specifically stimulated proliferation and differentiation of human TPOR-expressing cells and hematopoietic stem cells through stimulation of the PI3K, ERK, and STAT signaling pathways. It may represent a new small-molecule TPOR agonist which is orally active for patients with thrombocytopenia [69].

### 4.2. Experimental or Preclinical Agents

Some other drugs showed potential therapeutic ability for thrombocytopenia in preclinical research. TMEA is a natural chemical-structure small molecule which promoted MK differentiation through mTOR and ERK signaling and might be a novel TPOR agonist to treat thrombocytopenia [70]. Megakaryocyte differentiation is accelerated by 3,8-di-O-methylellagic acid 2-O-glucoside (DMAG) in vitro via the ERK/HIF1/NF-E2 pathway and it promoted platelet recovery in mice with thrombocytopenia. Therefore, DMAG is a promising drug for thrombocytopenia treatment [71]. The Chinese herbal medicine Caulis Polygoni Multiflori (CPM) can nourish blood, calm the mind, and treat anemia, and in vitro cell experiments showed that CPM significantly improved the differentiation and maturation ability of megakaryocyte cells. Animal experiments confirmed that CPM significantly accelerated megakaryopoiesis and platelet production in mice with radiation-induced thrombocytopenia and without resulting liver and renal toxicity. RNA-seq and Western blot results showed that CPM increased the expression of proteins involved in the MEK/ERK (MAPK) and PI3K/Akt signaling pathways. So, CPM is also a promising thrombopoietic agent for expanding clinical use [11]. Xanthotoxin (XAT) is another inhibitor that promotes megakaryocyte differentiation and maturation and **it** accelerates platelet production and recovery via activating the MEK/ERK signaling pathway, which provided a new pharmacotherapy treatment strategy for thrombocytopenia [72]. Alnustone promoted megakaryocyte differentiation and maturation in vitro, and restored platelet production in zebrafish and thrombocytopenic mice through MEK/ERK signaling, which also provided a new therapeutic strategy for thrombocytopenia treatment [73]. The miR-557 inhibitor improved ITP model rats’ symptoms with increasing the numbers of megakaryocytes and platelets which were detected by flow cytometry and Giemsa staining. Additionally, the miR-557 inhibitor upregulated the expression levels of p-ERK, p-Akt, and bcl-2 of TPO-induced megakaryocytes [36]. Juvenile myelomonocytic leukemia is splenomegaly with consequential thrombocytopenia in clinics. Most patients exhibit oncogenic mutations of PTPN11 (SHP2), leading to the hyperactivation of Erk and Akt. The combined treatment of p110δ and BTK inhibitors resulted in profound thrombocytopenia and anemia through strongly inhibiting the activation of both ERK and AKT. Therefore, BTK and PI3K as a dual-drug treatment are expected to be a new potential treatment for juvenile myelomonocytic leukemia-induced thrombocytopenia [52]. K562 cells treated with (R)-3-methoxy-3-oxo-2-stearamidopropyl phosphate (TEMOSPho) produced platelets and these platelets are functional; they expressed P-selectin and aggregated in response to AYPGFK and SFLLRN. (R)-TEMOSPho is a potential therapeutic drug for thrombocytopenia [24].

Many studies are conducted using animal models or in vitro systems. There are significant differences in signaling pathways between different species, both in vitro and in vivo. These experimental or preclinical inhibitors require further validation through clinical studies.

## 5. Conclusions

**Figure 1:**
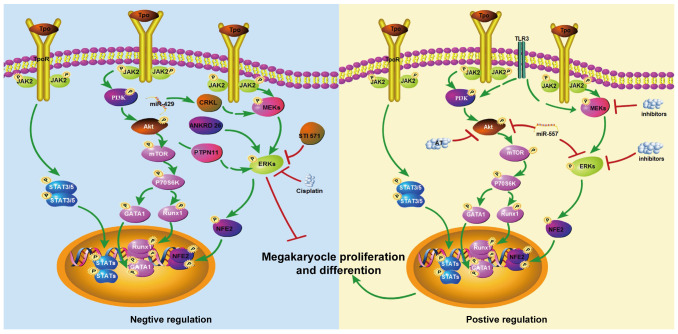
The opposed role of MAPK/ERK in megakaryopoiesis and differentiation. The MAPK/ERK signaling pathway is inhibited by STI571 and cisplatin, but activated by miR-429, ANKRD26, and PTPN11, resulting in decreased megakaryopoiesis and differentiation (as shown in the **left panel**); The MAPK/ERK signaling pathway is inhibited by miR-557, AT, and MEK or ERK inhibitors, resulting in increased megakaryopoiesis and differentiation (as shown in the **right panel**). Green arrows mean promotion effects and red T lines mean suppressive effects.

Thrombocytopenia is a common hematological disease. The MAPK/ERK signaling pathways have been reported to be involved in its pathogenesis through regulating megakaryocyte differentiation and platelet formation (Figure 1). Typical thrombopoietic agents targeting TPO-MAPK/ERK signaling are helpful for patient platelet recovery even with some side effects. Only a few agents are safe and effective to stimulate platelet production for thrombocytopenic patients in clinics, which makes the treatment options of thrombocytopenic patients limited. What’s worse, some types of thrombocytopenia have no available drugs to use, which increases the urgency of discovering novel mechanisms independent of TPO involving platelet production. Until now, only the model through gcForest reported on the ERK/HIF1/NF-E2 pathway which can be targeted by DMAG to promote megakaryocyte differentiation [71]. The gcForest drug screening model is a type of machine learning that offers high-throughput virtual screening of potential active compounds [74]. It improves the situation of traditional drug laboratory discoveries which are aimless and costly. Furthermore, the DMAG-induced ERK/HIF1/NF-E2 pathway is independently of the TPO signal, offering a novel strategy for drug screening in the treatment of hematologic diseases [71]. The MAPK/ERK signaling pathway still has big potential to be a target for thrombocytopenia treatment when we elucidate its role in thrombocytopenia patients with different situations. The expression level and sustainable time of the MAPK/ERK signaling pathway need to be checked and the stage of platelet formation or clearance time need to be tested. The understanding of platelet antigens and autoantibodies or immunocytes also needs to be strengthened.

## Figures and Tables

**Table 2:** The MAPK/ERK signaling pathway in tumor and tumor therapy-induced thrombocytopenia.

Disease Patterns	Signal Pathway	Correlation	References
Large granular lymphocyte leukemia-induced thrombocytopenia	MEK1/ERK pathway was activated	−	[49]
Chronic myeloid leukemia-induced thrombocytopenia	MEK1/ERK pathway was activated	−	[50]
I-MFA^−/−^ mice carcinoma model-induced thrombocytopenia	p-ERK signaling pathways were enhanced	−	[51]
Juvenile myelomonocytic leukemia-induced thrombocytopenia	Hyperactivation of ERK and AKT	−	[52,53]
JMML model mouse-induced thrombocytopenia	Hyperactive RAS/ERK signaling	−	[54]
The platelet count of murine lung carcinoma model was increased	p-AKT and p-ERK were decreasing	−	[55]
Hemolytic uremic syndrome induced thrombocytopenia	Activation of ERK, JNK and p38 MAPK signaling	−	[56]
Tesirofenib and sorafenib toxicities-induced thrombocytopenia	p-ERK was not inhibited	−	[57]
Navitoclax side effect induced thrombocytopenia in recurrent epithelial ovarian cancer	Not related to the expression of p-ERK	−	[58]
Cisplatin side effects induced thrombocytopenia	ERK pathway was significantly blocked	−	[59]

−: negative correlation.

**Table 3:** Drugs basis on the ERK/MAPK signaling pathway for the treatment of thrombocytopenia disease.

Drug Name	Status	Disease Patterns	Signal Pathway	References
Shengxuexiaoban Capsules	Launched	Primary ITP	PI3K-AKT and MAPK-ERK1/2	[67]
Ticlopidine	Launched	Thrombotic thrombocytopenic purpura	ERK1/2 and p38	[41]
Eltrombopag	Launched	Thrombocytopenia	AKT and ERK1/2	[27]
Eculizumab	Launched	Thrombocytopenia	the complement system	[68]
Hetrombopag	Launched	Thrombocytopenia	PI3K, ERK and STAT	[69]
TMEA	Preclinical research	Thrombocytopenia	mTOR and ERK signaling	[70]
DMAG	Preclinical research	Thrombocytopenia	ERK/HIF1/NF-E2	[71]
Caulis Polygoni Multiflori	Preclinical research	Radiation-induced Thrombocytopenia	MEK/ERK (MAPK) and PI3K/Akt	[11]
Xanthotoxin (XAT)	Preclinical research	Thrombocytopenia	MEK/ERK	[72]
Alnustone	Preclinical research	Thrombocytopenia	MEK/ERK	[73]
MiR-557 inhibitor	Preclinical research	ITP	p-ERK, p-Akt and bcl-2	[36]
BTK and PI3K dual drug	Preclinical research	Thrombocytopenia	ERK and AKT	[52]
(R)-TEMOSPho	Preclinical research	Thrombocytopenia	P-selectin expression and aggregated	[24]

## Data Availability

No new data were created or analyzed in this study. Data sharing is not applicable to this article.

## Abbreviations

The following abbreviations are used in this manuscript:

AKT	protein kinase B
aPL	antiphospholipid antibodies
APS	antiphospholipid syndrome
AT	atorvastatin
BTK	bruton tyrosine kinase
BVDV	the bovine viral diarrhea virus
c-MPL	c-mannosylation of thrombopoietin receptor
CIC	circulating immune complexes
CP	cytopathic
CPM	Caulis Polygoni Multiflori
DMAG	3,8-di-O-methylellagic acid 2-O-glucoside
ERK	extracellular signal-regulated kinase
GC	carboplatin
IL-2	interleukin-2
I-MFA	the inhibitor of MyoD Family A
JMML	juvenile myelomonocytic leukemia
MAPK	mitogen-activated protein kinase
MKs	megakaryocytes
mTOR	mammalian target of rapamycin
MVEC	microvascular endothelial cell
NCP	non-cytopathic
ITP	immune thrombocytopenia
p38	anti-P38 MAPK
PI3K	phosphatidylinositol 3-kinase
PMA	phorbol ester
RAS	activation of rat sarcoma virus
rhTPO	recombinant human thrombopoietin
(R)-TEMOSPho	(R)-3-methoxy-3-oxo-2-stearamidopropyl phosphate
SLE	systemic lupus erythematosus
STAT	signal transducer and activator of transcription
THC2	thrombocytopenia 2
TNFα	tumor necrosis factor α
TPOR	thrombopoietin receptor
TTP	thrombotic thrombocytopenic purpura
XAT	xanthotoxin
